# Proteoglycans in Biomedicine: Resurgence of an Underexploited Class of ECM Molecules

**DOI:** 10.3389/fphar.2019.01661

**Published:** 2020-01-29

**Authors:** Tanaya Walimbe, Alyssa Panitch

**Affiliations:** Laboratory of Engineered Therapeutics, Department of Biomedical Engineering, University of California, Davis, Davis, CA, United States

**Keywords:** proteoglycans, small leucine rich proteoglycans, decorin, fibromodulin, chondroitin sulphate, dermatan sulphate, heparan sulphate, extracellular matrix

## Abstract

Proteoglycans have emerged as biomacromolecules with important roles in matrix remodeling, homeostasis, and signaling in the past two decades. Due to their negatively charged glycosaminoglycan chains as well as distinct core protein structures, they interact with a variety of molecules, including matrix proteins, growth factors, cytokines and chemokines, pathogens, and enzymes. This led to the dawn of glycan therapies in the 20^th^ century, but this research was quickly overshadowed by readily available DNA and protein-based therapies. The recent development of recombinant technology and advances in our understanding of proteoglycan function have led to a resurgence of these molecules as potential therapeutics. This review focuses on the recent preclinical efforts that are bringing proteoglycan research and therapies back to the forefront. Examples of studies using proteoglycan cores and mimetics have also been included to give the readers a perspective on the wide-ranging and extensive applications of these versatile molecules. Collectively, these advances are opening new avenues for targeting diseases at a molecular level, and providing avenues for the development of new and exciting treatments in regenerative medicine.

## Introduction

As researchers try to harness the therapeutic potential of biopolymers for new treatments, proteoglycans (PGs) and their glycosaminoglycan (GAG) side chains remain underexploited due to their complex nature and involvement in multiple biological processes. Glycosaminoglycans are linear long chains of anionic glycan molecules that comprise one of the three major biopolymers found in the body, other than nucleic acids and proteins. GAGs are primarily made up of monomers of either glucuronic or iduronic acid and N-acetylglucosamine. These glycan monomers are not directly encoded by the genome and have a high degree of heterogeneity in terms of their monomer sequences, chain lengths, and sulfation patterns due to posttranslational modifications regulated in the golgi apparatus, leading to a large structural diversity with no defined glycan code (Hudak and Bertozzi, 2014). Six major types of GAGs are currently identified in mammals—chondroitin sulfate (CS), dermatan sulfate (DS), keratan sulfate (KS), heparan sulfate (HS), heparin (Hep), and hyaluronic acid (HA) (Köwitsch et al., 2018). Except for HA, all other GAGs are sulfated and exist as anionic molecules conjugated to core proteins, making them a component of proteoglycans (PGs). In addition to direct conjugation to core proteins, GAGs interact with other proteins through electrostatic or hydrophobic interactions, as well as hydrogen bonds, further adding to their broad repertoire and complexity.

PGs are a heterogenous family of macromolecules with 43 members, differing in their core protein as well as the nature and number of GAG chains bound to the core (Iozzo and Schaefer, 2015), reaching an unprecedented level of sophistication. These intriguing molecules have been conserved through millions of years of evolution to reach new heights of functional significances. Iozzo and Schaefer (2015) proposed a comprehensive classification and nomenclature for PGs based on their location, genetic homology, and use of protein modules. They classified PGs into four major classes with distinct forms and functions: Class 1 consists of intracellular secretory granules, class 2 consists of cell surface PGs that are classified as either transmembrane or GPI-anchored, class 3 consists of pericellular basement membrane zone PGs, and class 4 consists of extracellular PGs classified as hyalectan-lectincan (HA binding and lectin binding), spock, and small leucine rich PGs (SLRPs).

Early studies conducted on PGs focused on one of the PGs of the vertebrate cartilage extracellular matrix, now known as Aggrecan. Cartilage extracellular matrix is uniquely made with the majority of the non fibrillar ECM composition consisting of PGs and HA (Sophia Fox et al., 2009). PGs as structural components are known to hydrate, protect, and lubricate cartilage tissue (Lohmander, 1988); leading to a vast majority of therapeutics targeted to treating osteoarthritis harnessing these properties. Further PG research revealed that all cells in the body are covered by a gel like glycocalyx (Luft, 1966), which consists of PGs and GAGs involved in a myriad of signaling and growth factor sequestering activities (Weinbaum et al., 2007). Healthy endothelial glycocalyx is the only known blood contacting surface that prevents blood clotting continuously, due to the PGs in it creating a barrier for protein adsorption and fibrin formation. Altering of the glycocalyx has been implicated in various disease conditions (Tarbell and Cancel, 2016; Liew et al., 2017), making it a key target for development of therapeutics. Multifaceted functions of PGs are now known to include growth factor sequestering (Gubbiotti et al., 2016), providing adhesive properties, inducing or inhibiting angiogenesis (Järveläinen et al., 2015; Poluzzi et al., 2016), modulating cell adhesion, proliferation, and regulation (Christensen et al., 2019), as well as interacting with other ECM molecules and controlling collagen fibrillogenesis (Weber et al., 1996; Chen et al., 2010; Kalamajski and Oldberg, 2010). Researchers began recognizing the ubiquitous nature and essential functions of PGs, discovering their role as essential bioactive components of the ECM with sophisticated functions in maintaining homeostasis. However, PGs remained largely untapped as a class of potential therapeutics in comparison to recombinant antibodies and DNA technologies until the last decade. This lag in harnessing the potential of PGs is partially due to the complexity inherent to the synthesis, regulation, and assembly of these molecules. However, advances in carbohydrate biopolymer synthesis, recombinant technology, and the recognition of the enormous potential of PGs as treatments have led to an exciting reemergence of PG engineered therapeutics. PGs represent the most complex and multifunctional class of molecules, making them one of the most versatile and exciting classes of therapeutic candidates.

**Figure 1:**
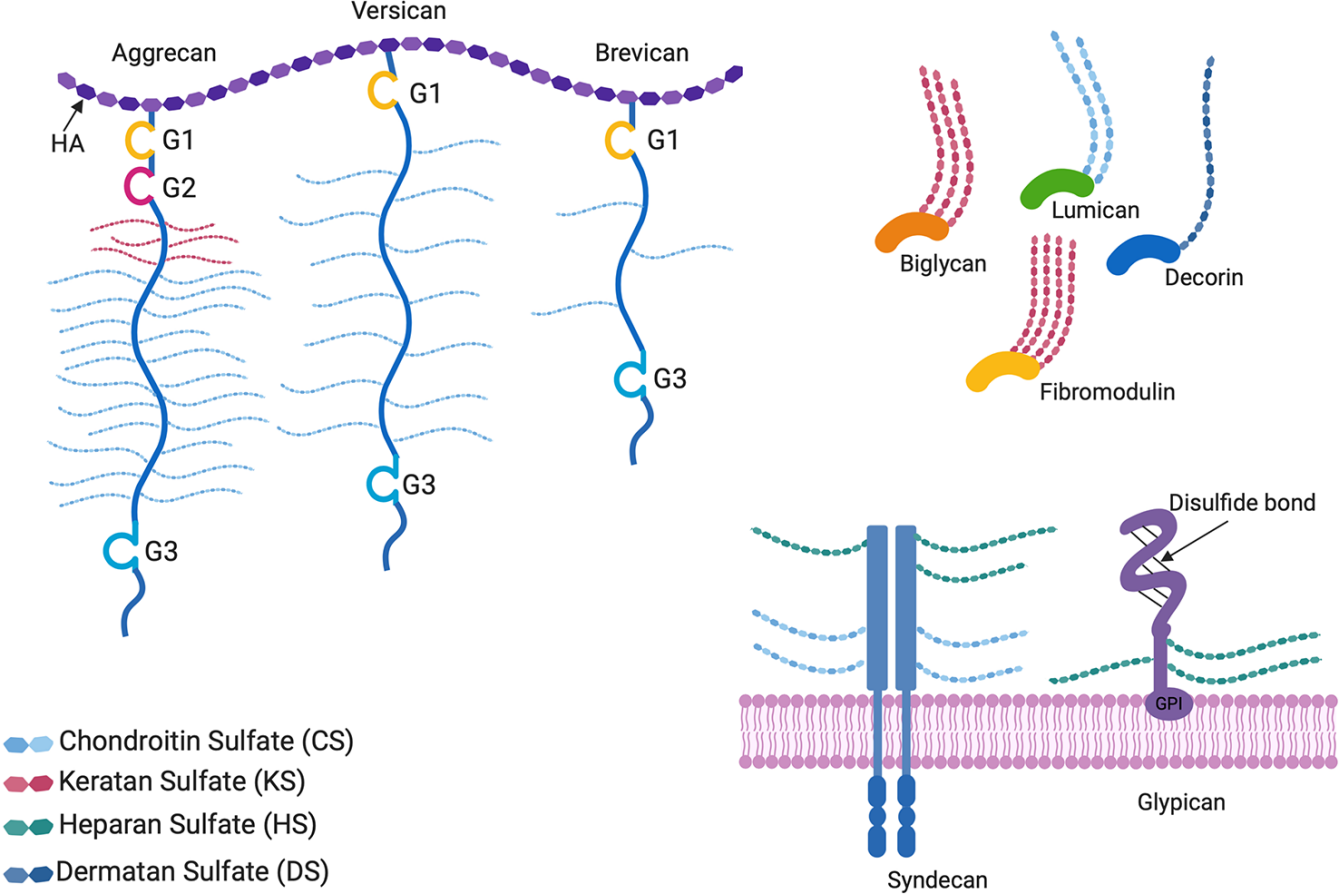
Proteoglycan structures. The horseshoe shaped SLRPs (decorin, biglycan, lumican, and fibromodulin) and bottlebrush structured hyaluronan (HA) binding proteoglycans (aggrecan, versican, and brevican) are located in the extracellular matrix, whereas glypicans and syndecans are cell surface proteoglycans. G1, G2, and G3 are globular structural domains located at the N- and C-terminus of HA binding proteoglycan cores. Glypicans are bound to the cell surface by glycophosphatidylinositol (GPI) anchors. All proteoglycans differ in the GAG side chains attached to the core protein, as well as the lengths and sulfation patterns of the GAGs, thus adding to their complexity.

The focus of this review is limited to recent advances and preclinical studies on naturally occurring proteoglycan molecules and proteoglycan mimetics as ECM based therapeutics. Built on decades of information about the complex signaling pathways and their downstream effectors, scientists are using PG core proteins, glycanated PGs, neo-PGs and PG mimetics to tackle human health and disease. To stay within the scope of the review, developments on GAGs alone as therapeutics, or detailed descriptions of the complex functions of all proteoglycans were not included. In order to provide context for harnessing the therapeutic value of PGs, structures of common proteoglycans are depicted in **Figure 1**. Readers are directed to read about the current developments in the use of GAGs alone as glycan therapeutics in Paderi and coworkers’ recent review, which discusses the clinical relevance, applications and clinical stage pharmaceutical developments of these entities (Paderi et al., 2018). For in-depth information about the biological functions of PGs, readers are encouraged to read Izzo and Schaefer’s recent review (Iozzo and Schaefer, 2015).

## Cell Surface PGs

### Glypicans

Glypicans are heparan sulfate proteoglycans (HSPGs) that are bound to the cell surface by glycophosphatidylinositol (GPI) anchors (see **Figure 1**). Glypican 1 nanoliposomes have been used to potentiate therapeutic angiogenesis for ischemic wound healing by the Baker group (Monteforte et al., 2016). Co-delivery of glypican-1 with FGF-2 markedly increased the recovery of perfusion and vessel formation in ischemic hind limbs of wild type and diabetic mice in comparison to mice treated with FGF-2 alone, proving that the proteoglycan played an important role in potentiating the activity of FGF-2. Han et al. (2016) showed that lower levels of glypican-3 were detected in patients with gastric cancer than in healthy gastric tissue, showing an inverse correlation between GP-3 levels and metastasis. Targeting glypican-3 or its downstream signaling pathways, or supplementation with adenoviral overexpression of glypican-3 in such cases might therefore, have the potential to suppress metastasis related to gastric cancer.

### Syndecans

Syndecans are also HSPGs that act as transmembrane receptors capable of signaling independently or in combination with other receptors and integrins (Morgan et al., 2007; Elfenbein and Simons, 2013). Das and coworkers from the Baker research group have researched the use of syndecan 4 for the treatment of diabetic wound healing because of FGF2 coreceptor activity of syndecans (Elfenbein and Simons, 2013). Syndecan 4 encapsulated in proteoliposomes as a delivery system showed promise in the treatment of diabetic ischemia in mice (Das et al., 2016a). Co-therapy of Syndecan with FGF-2 successfully enhanced therapeutic angiogenesis and sustained revascularization in the ischemic hind limb of diabetic, obese mice in comparison to the use of FGF2 alone (Das et al., 2016b). They also went on to test the effects of these proteoliposomes on PDGF-BB activity (Das et al., 2016b). Wounds treated with both syndecan-4 proteoliposomes and PDGF-BB had increased re-epithelization and angiogenesis in comparison to wounds treated with PDGF-BB alone. Moreover, the wounds treated with syndecan-4 proteoliposomes and PDGF-BB also had increased M2 macrophages and reduced M1 macrophages, suggesting syndecan-4 delivery induces immunomodulation within the healing wounds. These results demonstrate the promise of proteoglycans, in particular syndican-4, as a co-therapy for tissue regeneration and the treatment of nonhealing wounds.

Many glycan–protein interactions take place at the cellular interface, and cell surface PGs, especially syndicans, are heavily involved in growth factor interactions and cellular response to wound healing (Gallo et al., 1996; Brooks et al., 2012; Elfenbein and Simons, 2013). Given importance of membrane bound glycans including the syndicans and glypicans, future work to address key mechanistic queries such as whether the liposomes containing cell surface PGs fuse with cells, or is the mere presence of HS near the membrane surface a key factor in driving therapeutic potential at the cellular interface, could drive key developments in this burgeoning field.

## Lecticans

### Aggrecan

Aggrecan is of great interest to many researchers due to its load bearing and water retaining ability to protect and hydrate cartilage tissue. Aggrecan exhibits a bottlebrush like structure in which chondroitin sulfate and keratan sulfate GAG chains are attached to a core protein consisting of 3 globular structural domains (see **Figure 1**) (Kiani et al., 2002). The Marcolongo group has done extensive characterization of bottle brush polymers using chondroitin sulfate and other synthetic polymers (instead of a core protein) as core-bristle aggrecan mimetics. By establishing a method to functionalize chondroitin-4-sulfate at the reducing end and incorporating it into either poly(acrylic acid) (PAA) or poly(acryloyl chloride), they were able to achieve large 1.6 MDa polymers with enhanced water uptake as compared to aggrecan alone. By modulating the size of the PAA and number of CS chains bound to it, they were able to successfully form polymers with tunable osmotic pressures for the treatment of osteoarthritis. These mimetics were also shown to diffuse through the cartilage matrix into the pericellular area, and integrate into rabbit tissue before and after static loading, demonstrating the ability to engineer ECM on a molecular level (Sarkar et al., 2012; Prudnikova et al., 2017; Prudnikova et al., 2018; Phillips et al., 2019).

The Kipper research group designed graft copolymer nanoparticles of cationic polysaccharides such as chitosan with anionic GAGs like CS and Hep to form polyelectrolyte complex nanoparticles mimicking the size and chemistry of aggrecan. These nanoparticles were shown to maintain FGF-2 activity after 21 days of encapsulation and are superior to aggrecan alone as a delivery vehicle for growth factors (Place et al., 2014b). In a separate study to mimic PGs, they also generated copolymers using a heterobifunctional crosslinker to combine HA to the reducing ends of Hep/CS. They also reported successful grafting of these polymers to chitosan for the delivery of FGF2 (Place et al., 2014a).

Our lab has taken a different approach by mimicking the function of aggrecan, but not its structure. We have designed an aggrecan mimetic that is composed of chondroitin sulfate decorated with HA-binding peptides in an effort to mimic key aggrecan function for the treatment of osteoarthritis. These aggrecan mimetics have been shown to penetrate aggrecan-depleted cartilage, contribute to its overall compressive strength, and reduce catabolic activity in *in vivo* and *ex vivo* models of osteoarthritis (Bernhard and Panitch, 2012; Sharma et al., 2013; Sharma et al., 2016). Unlike hyaluronan and chondroitin sulfate, these mimetics were able to promote type II collagen synthesis and aggrecan expression when encapsulated with bovine chondrocytes in collagen hydrogels. The mimetic was also shown to be resistant to the enhanced proteolytic activity found in OA cartilage, since it lacks the known aggrecan cleavage sites.

From the above studies, two major approaches stand out in efforts to harness the biological activity of aggrecan—mimicking its structure, vs targeting GAGs to tissue locations; for example, by targeting HA binding to augment surrounding ECM. Both approaches have extensive potential to achieve improved tissue function and healing. Clinical applications for osteoarthritis treatment using these PG mimicking polymers appear on the near horizon as advanced synthesis and scale up techniques for protein conjugation and polymer synthesis become more readily available.

## Small Leucine Rich PGs

By far the most widely researched class of PGs, SLRPs share structural similarities in their core protein of leucine rich tandem repeats flanked by cysteine rich repeats. The biological functions of SLRPs are too vast to be summarized in a single review, hence, we refer readers to recent comprehensive reviews focusing specifically on SLRPs (Iozzo, 1997; Iozzo, 1999; Schaefer and Iozzo, 2008; Chen and Birk, 2013; Hultgårdh-Nilsson et al., 2015; Nastase et al., 2018; Appunni et al., 2019). It is widely accepted that the horseshoe-shaped core protein of SLRPs is responsible for its binding to collagen, modulating collagen fibrillogenesis and protecting collagen from enzymatic cleavage (Karamanos et al., 2018). As new information comes to the forefront of SLRP research, researchers have discovered that the functionality of SLRPs changes based on whether the core protein is attached to its GAG chains, or as unmodified core protein (Yu et al., 2018). Multiple forms of these PGs are thus used as therapeutic candidates.

### Decorin

Decorin is the archetypal, most extensively studied SLRP, and has been vastly characterized for its influence on collagen fibrillogenesis (Danielson et al., 1997) and involvement in scarless wound healing. Decorin is not just a structural entity, it plays a pivotal biological role in angiogenesis (Järveläinen et al., 2015), inflammation (Nastase et al., 2018), fibrosis (Ahmed et al., 2014), wound healing (Grisanti et al., 2005), oncosuppression (Sainio and Järveläinen, 2019), and endothelial cell health and autophagy (Neill et al., 2017) to name some. Due to this involvement in an enormous range of biological functions, decorin has aptly been termed as a “guardian from the matrix” (Neill et al., 2012). Decorin consists of a core protein with small leucine rich tandem repeats, with a dermatan sulfate or chondroitin sulfate GAG chain attached to it through the N terminus of the protein. Through its GAG side chain and core protein, it can bind to various growth factors such as TGFβ, as well as collagen and other ECM molecules, whereby it likely serves as a reservoir for TGFβ and stabilizes inter fibrillar organization of the collagen (Orgel et al., 2009).

Since the invention of human recombinant decorin core protein expressed in CHO cells, this PG has been manufactured using cGMP conditions and is being tested as a therapeutic for multiple disease indications, arguably bringing it closest to clinical implementation. Galacorin, the trademark name for the decorin drug produced through Catalent pharma, is being tested for the treatment of macular degeneration, diabetic retinopathy, and diabetic macular edema (Devore et al., 2010).

From a therapeutic research perspective, decorin has been investigated for its use in corneal wound healing. Grisanti et al. used decorin in an experimental glaucoma filtration surgery pilot study on rabbits (Grisanti et al., 2005). Postoperative results showed that rabbits treated with decorin had significantly less ECM deposition 14 days after surgery, as well as suppressed conjunctivital scarification. Hill et al. (2018) designed gellan based fluid gels for sustained delivery of human recombinant decorin through eye drops for corneal regeneration and found improved ocular function. Due to its ability to delay collagen fibrillogenesis, decorin is an attractive therapeutic candidate for anti-scarring treatments. It also acts as a TGF- β1/2 antagonist, and has been used as a treatment against spinal scarring. Ahmed et al. (2014) showed that treatment of acute and chronic dorsal funicular spinal cord lesions (DFL) in adult rats with decorin resulted in a reduction in wound cavity area, suppression of inflammatory fibrosis, and dissolution of mature scars due to decorin’s fibrolytic activity and neutralization of TGF- β1/2. In an independent study, decorin treatment reduced hypertrophic scarring through inhibition of the TGF-β1/Smad signaling pathway in a rat osteomyelitis model (Wang et al., 2016).

Decorin is also considered a potent oncosuppresor due to its ability to function as an endogenous pan‐receptor tyrosine kinase inhibitor, a regulator of both autophagy and mitophagy, as well as a modulator of the immune system (Ahmed et al., 2014). Oncolytic adenovirus expressing decorin significantly inhibited the progression of bone metastases in MDA-MB-231 metastasis model of breast cancer (Yang et al., 2015). Adenovirus overexpression of IL-12 and decorin have demonstrated potent antitumor effects in a weakly immunogenic murine model of breast cancer (Oh et al., 2017a). Along similar lines, adenoviral overexpression of decorin and Granulocyte Macrophage Colony Stimulating Factor has shown anti-tumor potential in a model of murine colorectal cancer (Liu et al., 2017) (Wang et al., 2016). Shen and coworkers engineered a recombinant decorin fusion protein with an extended C-terminus comprised of a vascular homing peptide that recognizes inflamed blood vessels and penetrates deep into the vessel wall, known as CAR. In a study to evaluate its efficacy as a treatment for abdominal aortic aneurysm (AAA), they delivered the CAR-DCN molecule to mice with angiotensin-II induced AAAs, and found increased 28 day survival and reduced severity of AAA post treatment (Shen et al., 2017).

In addition to using the native decorin core protein and GAG-decorated molecules, synthetic mimetics of decorin have been developed. In an effort to mimic the collagen modulating function of decorin, our lab has designed a decorin mimetic made of collagen-binding peptides conjugated to a dermatan sulfate backbone (Paderi and Panitch, 2008). Similar to decorin, this molecule influences the fibril diameter of type I collagen on a nanoscale. Stuart et al. showed that these mimetics reduce dermal scarring in a rat linear incision model, due to their ability to mask existing collagen from matrix metalloprotease (MMP-1 and MMP-3) mediated proteolytic degradation while modulating collagen organization (Stuart et al., 2011). In addition, it was reported that similar to the anticoagulant, anti-thrombotic function of the glycocalyx, this mimetic was able to bind to exposed collagen in denuded arteries within minutes to suppress platelet binding and activation, and thus prevent resulting vascular intimal hyperplasia that would normally occur after percutaneous coronary intervention (PCI) sans mimetic (Scott and Panitch, 2014; Scott et al., 2017). The mimetic, termed DS-SILY, was able to reduce smooth muscle cell proliferation and migration, as well as reduce intimal hyperplasia *in vivo* in Ossabaw pigs by 60% as compared to controls (Paderi et al., 2011; Scott et al., 2013). After extensive *in vitro* and *in vivo* validation, DS-SILY is licensed through Symic bio, and is being tested in clinical trails for the treatment of peripheral vascular disease.

### Lumican

Lumican has been extensively studied as a keratan sulfate proteoglycan responsible for corneal transparency and wound healing. Like other SLRPs, it is involved in modulating collagen fibrillogenesis and interacts with growth factors through its core protein (Rada et al., 1993). *Lum*(–/–) knockout mice have given way to an enormous amount of research diving into the unique functions of lumican in tendon and skin health, and corneal transparency (Chakravarti, 2002). The Chakravarti group has used these knockout mice to bring forth the importance of lumican in various indications such as bacterial phagocytosis, innate immunity, and corneal clearing. In a mouse corneal Lum(–/–) model infected with *Pseudomonas aeruginosa*, lumican was shown to be responsible for bacterial clearing and facilitation of an innate immune response. In *P. aeruginosa* lung infections, lumican-deficient Lum(–/–) mice failed to clear the bacterium from lung tissues, and showed poor survival rates (Shao et al., 2013a). Lumican modulates wound healing and innate immunity by interacting with receptors and immune cells such as macrophages (Shao et al., 2013b).

Soluble lumican core protein isolated from human amniotic membranes has been shown to effectively promote epithelial proliferation and migration in a study by Yeh et al. (2005). Lumican modulates fibroblast contact through the α2β1 integrin, a finding that has been exploited for therapeutic development. Recombinant lumican application on mice skin wounds showed enhanced wound healing in a study by Liu et al. (2013), possibly due to lumican promoting the contractility of fibroblasts through the α2β1 integrin. In an independent study, adenoviral overexpression of lumican in hypertrophic scarring rabbit models and fibroblasts effectively thinned the scar area and inhibited fibroblast proliferation, as well as successfully reduced focal adhesion kinase (FAK) phosphorylation as a result of binding to α2β1 integrin (Zhao et al., 2016).

Gesteira et al. (2017) designed a peptide mimicking the activity of lumican based on 13 C-terminal amino acids of lumican (LumC13). They showed that the peptide effectively forms a complex with type I receptor for TGFβ1 (ALK5) and promoted corneal wound healing in mice (Yamanaka et al., 2013). Lumican derived peptides–lumcorin, have been tested against melanoma and show therapeutic potential by inhibiting cell chemotaxis and melanoma growth through MMP-14 inhibition (Zeltz et al., 2009; Pietraszek et al., 2013). The vast array of studies showing the biological activity of lumican underscore the importance of proteogycans in homeostasis and disease and highlight the potential of targeting these ECM molecules to treat disease.

### Biglycan

Biglycan shares structural similarities with decorin and comprises 12 leucine-rich repeats flanked by cysteine-rich domains. It is a major component of bone, cartilage, tendon and muscle. Biglycan has been studied as a potential therapeutic for musculoskeletal disorders, due to its involvement in modulating collagen fibrillogenesis as well as its role in modulating and maintaining musculoskeletal organization (Young and Fallon, 2012). Biglycan is predominantly expressed as a proteoglycan, but a mature form lacking GAG side chains, known as “nonglycanated” biglycan, has recently been shown to have specific functions in muscle and Wnt signaling (Amenta et al., 2011).

Duchenne muscular dystrophy (DMD) is caused by the loss of dystrophin in muscles, leading to membrane fragility and impaired signaling. Non-glycanated recombinant biglycan delivered to dystrophic mice has been shown to recruit utrophin, an autosomal paralog of dystrophin, and a NOS-containing signaling complex to the muscle cell membrane to improve muscle health and function (Amenta et al., 2011). In an independent follow up study, Ito et al. hypothesized that biglycan expressed in a small number of muscle fibers was likely to have been secreted and anchored to the cell surface throughout the whole muscular fibers to improve motor function (Ito et al., 2017). An optimized version of the nonglycanated biglycan, “TVN-102”, is under development as a candidate therapeutic for DMD (Fallon and McNally, 2018).

### Fibromodulin

Fibromodulin (Fmod) has been widely investigated for its role in fetal-like scarless wound healing and angiogenesis. The Soo research group demonstrated that Fmod stimulated capillary infiltration into Matrigel plugs, enhanced angiogenesis in chick chorioallantoic membrane (CAM) assays, and restored the vascularity of *fmod^−/−^* mouse wounds (Jian et al., 2013; Zheng et al., 2014). These results suggest enhanced angiogenesis during cutaneous wound healing, proving that Fmod is an attractive therapeutic candidate for wound management especially in cases where angiogenesis is impaired, such as diabetic wounds. They also went on to use Fmod to reprogram fibroblasts into a multipotent cell type as a means to bypass mutation and malignancy risks associated with genetically modified iPS cells (Zheng et al., 2012; Li et al., 2016). Testing these reprogrammed cells *in vitro* and in a clinically relevant critical-sized calvarial defect model, they demonstrated strong osteogenic capacity of these cells without tumorigenesis, showing that Fmod reprogrammed cells present potential for bone regeneration.

Adenoviral transfection of fibromodulin (ad-Fmod) has gained popularity in the past decade, and multiple studies have utilized ad-Fmod to target wound healing and cancer. Jazi et al. (2016) probed the therapeutic effects of recombinant adenoviral vectors expressing Fmod for the treatment of diabetic nephropathy in streptozotocin induced diabetic rats. They found reduced expression of TGFβ1 in rats transfected with Fmod gene transfer, suggesting a mechanism of action for fibromodulin therapy. Given its potent role in promoting angiogenesis and wound healing, a study by Ranjzad et al. (2009) demonstrated significant reduction in neointimal thickness and area in an *ex vivo* human saphenous vein organ culture model following adenovirus mediated fibromodulin gene transfer. Delalande et al. (2015) used non-viral histidylated vectors for Fmod gene transfer and local Fmod expression to enhance achilles tendon healing; they demonstrated promising improvements in biomechanical and histological parameters in a rat achilles tendon injury model. Fmod has been shown to successfully inhibit the nuclear factor-κB (NF-κB) signaling and induce fibroblast apoptosis (Lee and Schiemann, 2011). Dawoody Nejad et al., 2017 demonstrated that recombinant Fmod was able to suppress TGFβ1 and NF- κB activity *in vitro* in a highly metastatic breast cancer cell line (Dawoody Nejad et al., 2017). 

In summary, it is evident from the wide body of research reviewed above, that SLRP core proteins and their GAG components have important, and sometimes distinct, activities. The numerous approaches to use recombinant core proteins and functional mimetics highlight the diversity of strategies that can be employed in the use of SLRPs to enhance tissue regeneration and wound healing. To learn more about the recombinant production of PGs and their different domains, readers are encouraged to read Lord and Whitelock (2013) concise review. Further work in this field is warranted to better delineate the biological function of the core proteins, GAGs and synergies of the two to design therapies that focus on cell-ECM interactions, and are effective on a molecular level.

## Other PGs

### Proteoglycan 4/Lubricin

Proteoglycan 4 (PRG4) or lubricin is a mucin like proteoglycan/glycoprotein found in the synovial fluid of cartilage. It is responsible for lubricating the surface boundary of cartilage in synergy with HA. Interestingly, inflammation and osteoarthritis progression show an inverse relationship to lubricin expression, suggesting that it is directly involved in reducing inflammation and boundary friction levels (Iqbal et al., 2016).

Exploiting this information, the Schmidt and Tannin groups have extensively shown that PRG4 supplementation can restore normal cartilage boundary lubrication function to osteoarthritic SF (Schmidt et al., 2007; Ludwig et al., 2012). They have since, established a method for recombinant lubricin production, and are testing the functional effects of lubricin in other therapeutic areas such as intraabdominal lesions and contact lenses for ocular applications (Oh et al., 2017b; Samsom et al., 2018b). Lubris biopharma is a clinical stage start up company that is testing human recombinant lubricin for the treatment of dry eye (Lambiase et al., 2017) and osteoarthritis due to its role as a boundary lubricant. In an independent study by Larson et al. (2016), recombinant lubricin effectively reduced the coefficient of friction of bovine cartilage explants inflamed using IL1β. This further adds to the body of literature displaying the potential of lubricin in the treatment of osteoarthritis.

We and others have taken an approach that mimics the lubricating function of lubricin, but not its structural properties. We have designed a lubricin mimetic (mLub) by attaching type II collagen and HA binding peptides to a chondroitin sulfate backbone. Work done by Lawrence et al. (2015) demonstrated the ability of the mimetic to bind to articular cartilage and reduce the coefficient of friction on a macroscale. The Grinstaff lab designed anionic hydrophilic bottle-brush polymer lubricants using poly(7-oxanorbornene-2-carboxylate) as biolubricants for the treatment of osteoarthritis (Wathier et al., 2010). Synthesized *via* ring-opening metathesis polymerization, the polymer biolubricant showed promise in reducing friction and offering chondroprotection in *ex vivo* plug-on-plug and rat models of osteoarthritis (Wathier et al., 2013; Wathier et al., 2018). Further efforts to improve the polymer to make it better match the osmolarity of synovial fluid are being conducted by making it less anionic and covalently conjugating pendent triethylene glycol (TEG) chains to it (Lakin et al., 2019). A note about lubricin—it is debated whether this molecule is a glycoprotein or a proteoglycan, since it is a glycosylated protein that does not have traditional glycans such as CS, DS, or heparin attached to a protein core, and the protein itself is glycosylated.

### Perlecan (Heparan Sulfate Proteoglycan 2)

Perlecan is a large HSPG with a protein core composed of five distinct domains, which impart it with a wide range of functionalities to interact with other biological molecules (Douglass et al., 2015). The GAG-bearing domain I of Perlecan has been shown to promote chondrogenesis (French et al., 2002). Using this information, researchers have synthesized hydrogels containing the perlecan domain I along with HA (Jha et al., 2009) or type II collagen (Yang et al., 2006) to demonstrate enhanced binding and activity of bone morphogenic protein 2 (BMP2). BMP2 is considered a primary stimulant of chondrogenesis, and both studies showed robust stimulation of a cartilage specific ECM in comparison to controls not containing perlecan. Additionally, injectable microgels made up of HA and perlecan domain I showed enhanced activity of BMP2 in promoting cartilage matrix synthesis in a mouse early osteoarthritis model (Srinivasan et al., 2012). These results demonstrate that combining specific PG domains with hydrogels to drive growth factor activity may provide a higher level of control over cell fate and disease modulation.

Primarily considered a proangiogenic molecule, perlecan interacts with FGF2 and VEGF to regulate angiogenesis (Aviezer et al., 1994; Zoeller et al., 2009), making it an attractive potential therapy for wound healing where angiogenesis is impaired. Domain V (DV) of perlecan has been heavily investigated for its role in angiogenesis. The Bix group has investigated the potential of DV of perlecan to counteract the effects of amyloid-β (Aβ), which causes neurovascular dysfunction (Parham et al., 2014). Results from their studies showed improved endothelial proliferation, migration, and tubule formation despite treatment with Aβ by directly interfering with the α2 and α5 integrins (Clarke et al., 2012; Parham et al., 2016), thus promoting angiogenesis and supporting DV’s potential as an anti-amyloid therapeutic. Among other neurovascular applications, DV has been suggested as a potential treatment for stroke and vascular dementia (Marcelo and Bix, 2015). A study by Lee et al. (2011) demonstrated enhanced post-stroke angiogenesis in rat and mouse models of stroke after DV treatment, suggesting it as a neuroprotective approach for stroke treatment. As a strategy to develop bioactive vascular grafts, Rnjak-Kovacina et al. (2016) functionalized silk with perlecan DV decorated with heparan sulfate and chondroitin sulfate chains to enhance endothelial cell adhesion and proliferation while inhibiting platelet binding effectively. These studies highlight the complicated balance between proteoglycan activity with and without their attached sidechains and emphasize the implications of these variations in therapeutic developments.

### NeoPGs

The Godula research group synthesized HSPG neoPGs that completely circumvented the limitations of HSPG synthesis such as heterogeneity and batch to batch variability by incorporating disaccharides (diGAGs) generated by depolymerization of HS by bacterial heparinases into a poly(acrylamide) scaffold decorated with pendant *N*-methylaminooxy groups, which are reactive toward the hemiacetal functionality of the reducing glycans (Huang et al., 2014). By synthesizing a library of neoPGs and designing a microarray for testing binding to FGF-2, they were able to shortlist neoPGs with affinity to FGF2, which showed enhanced promotion of neural specification in embryonic stem cells deficient in HS biosynthesis.

The Hsieh-Wilson lab specializes in synthesis of glycomimetics to research the influence of GAG position and density on their avidity and specificity to interact with other proteins. Using end-functionalized ring-opening metathesis polymerization (ROMP) based polymers that mimic the native-like, multivalent architecture found on chondroitin sulfate (CS) proteoglycans, Lee et al. (2010) used norborene based backbones with biotin functionalized pendant sugars to create glycomimetics of various molecular weights and sulfation motifs. By controlling the sulfation patterns and display of these pendant sugars, novel mimetics for CS proteoglycans can be designed for targeted regeneration (Sotogaku et al., 2007; Miller and Hsieh-Wilson, 2015; Stopschinski et al., 2018).

The Pashkuleva group has designed mimimalistic PG mimetics by coassembly of aromatic peptides and carbohydrate amphiphiles. The amphiphiles Fmoc-glucosamine-6-sulfate (GlcN6S) and Fmoc-glucosamine-6-phosphate (Fmoc-GlcN6P) provided the functional element through the sulfate and phosphate groups, while fluorenylmethoxycarbonyl-diphenylalanine (Fmoc-FF) acted as a structural component, forming self-sustained macroscopic gels that are biocompatible and mimic the PG growth factor sequestering action, making these gels attractive for tissue engineering applications (Brito et al., 2019). In a separate study, Novoa-Carballal et al. (2018) synthesized star-like PG mimetics by grafting high molecular weight GAGs such as heparin and CS to hyperbranched synthetic cores like polyglycerol using oxime condensation. These mimetics showed enhanced binding to proteins by forming microfiber complexes instead of spherical nanocomplexes that form with linear GAGs, thus showing a larger degree of potential for modulating protein activity and presentation.

The Hudalla group is focused on creating self-assembling beta sheet nanofibers using synthetic glyco-peptides as supramolecular mimetics of glycoproteins. Hydrogels formed by these glycopeptides contain decorated n-acetylglucosamine and n-acetyllactosamine residues, which impart the gels with avidity to various proteins, especially galectins, a carbohydrate binding class of proteins involved in modulating cell proliferation, adhesion and apoptosis (Restuccia et al., 2015; Restuccia and Hudalla, 2018). By optimizing the content of n-acetyllactosamine residues, they aim to inhibit protein-glycan interactions implicated in autoimmune and cancer disease progressions.

**Figure 2:**
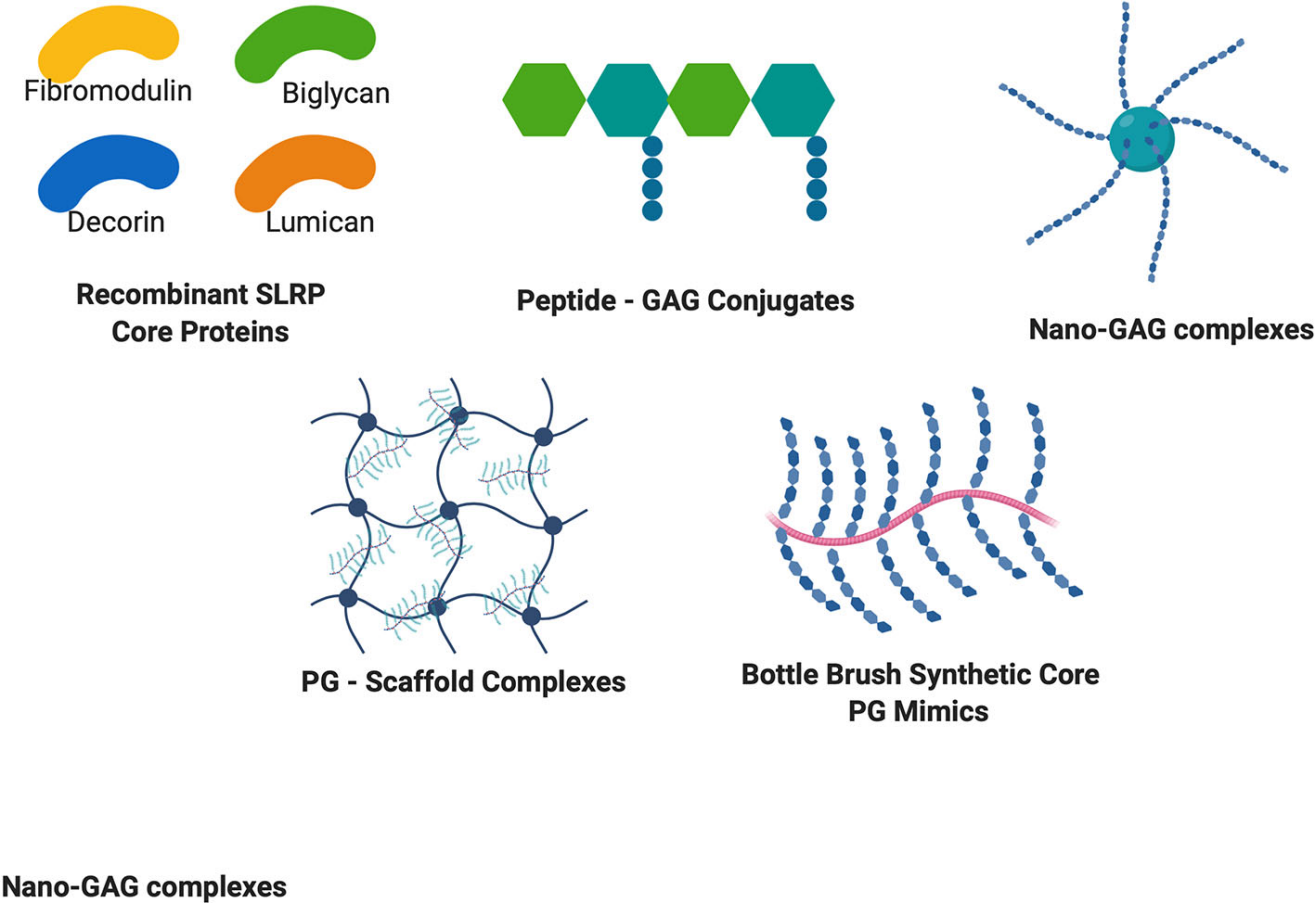
Structures of recombinant PG cores and PG mimetics. Recombinant production of PG cores and variants in many cell lines has accelerated discovery of the therapeutic potential of PGs. Peptide-GAG conjugates and nano-GAG complexes are being used to mimic PG functions and binding ability. PG – Scaffold complexes are being explored as tissue engineering constructs for regenerative medicine, and bottle brush mimetics of PGs such as aggrecan are being explored to circumvent increased degradation of PGs to provide enhanced healing potential.

Overall, these synthetic approaches summarized in **Figure 2** bring new knowledge on structure function relationships as well as a powerful approach to design cell-ECM interactions to improve tissue function and healing.

## Conclusions and Perspectives

While pioneering researchers have been focused on the roles of GAGs and proteoglycans for years, it has only been within the last two decades that the staggering potential of PGs to modulate tissue environments has been more broadly appreciated. Their multifunctional biological processes, in particular, their ability to bind and sequester growth factors and interact with various ECM molecules and influence cellular signaling events, makes them extremely attractive drug conjugates for multiple disease indications. **Table 1** summarizes common proteoglycans and their therapeutic applications.. Clinical translation of these molecules, however, remains a challenge. Due to the advent of recombinant technology, adenoviral and non-viral gene transfers are attractive alternatives to purifying native PGs, a task that is considered extremely difficult and time intensive. However, while recombinant technology can synthesize core proteins of PGs fairly consistently, their post-translational GAG chain modifications remain a challenge. Some GAG chain structures require enzymes in the Golgi apparatus only found in mammals, and absent in single celled organisms used to synthesize recombinant PGs. Effectively conveying the mechanism of action of these drugs also remains a significant challenge, due to the diverse processes with which these molecules interact.

**Table 1:** Summary of proteoglycans and their therapeutic applications.

Proteoglycans	Predominant GAG	Therapeutic Application	References
**Glypican 1-6**	Heparan sulfate	Ischemic wound healing	(Monteforte et al., 2016)
		Suppressing metastasis in gastric cancer	(Han et al., 2016)
**Syndecan 1-4**	Heparan sulfate	Diabetic wound healing	(Das et al., 2016a; Das et al., 2016b; Das et al., 2016c)
**Aggrecan**	Chondroitin sulfate, keratan sulfate	Osteoarthritis	(Bernhard and Panitch, 2012; Place et al., 2014a; Phillips et al., 2019; Place et al., 2014b; Sarkar et al., 2012; Sharma et al., 2013; Sharma et al., 2016; Prudnikova et al., 2017; Prudnikova et al., 2018)
**Decorin**	Dermatan sulfate	Macular degeneration, diabetic retinopathy, diabetic macular edema	(Devore et al., 2010)
		Corneal wound healing	(Grisanti et al., 2005; Hill et al., 2018)
		Anti-scarring	(Stuart et al., 2011; Ahmed et al., 2014; Wang et al., 2016)
		Oncosupression	(Yang et al., 2015; Wang et al., 2016; Liu et al., 2017; Oh et al., 2017a)
		Abdominal aortic aneurysm	(Shen et al., 2017)
		Vascular neointimal hyperplasia	(Paderi et al., 2011; Scott and Panitch, 2014; Scott et al., 2017)
**Lumican**	Keratan sulfate	Corneal wound healing	(Chakravarti, 2002; Gesteira et al., 2017)
		Bacterial lung infections	(Shao et al., 2012; Shao et al., 2013a)
		Scarring	(Yeh et al., 2005; Liu et al., 2013; Yamanaka et al., 2013; Zhao et al., 2016)
		Melanoma	(Zeltz et al., 2009; Pietraszek et al., 2013)
**Biglycan**	Chondroitin sulfate	Duchenne muscular dystrophy	(Amenta et al., 2011; Ito et al., 2017; Fallon and McNally, 2018)
**Fibromodulin**	Keratan sulfate	Diabetic wounds and neuropathy	(Jian et al., 2013; Zheng et al., 2014; Jazi et al., 2016)
		Neointimal hyperplasia	(Ranjzad et al., 2009)
		Bone regeneration	(Zheng et al., 2012; Li et al., 2016)
		Tendon healing	(Delalande et al., 2015)
		Breast cancer metastasis	(Dawoody Nejad et al., 2017)
**Lubricin**	None	Osteoarthritis	(Iqbal et al., 2016; Lakin et al., 2019; Larson et al., 2016; Ludwig et al., 2012; Wathier et al., 2013; Lawrence et al., 2015)
		Ocular applications, dry eye	(Lambiase et al., 2017; Oh et al., 2017b; Samsom et al., 2018a)
**Perlecan**	Heparan sulfate	Cartilage regeneration	(French et al., 2002; Yang et al., 2006; Jha et al., 2009; Srinivasan et al., 2012)
		Ischemic wound healing	(Aviezer et al., 1994; Zoeller et al., 2009)
		Neurovascular dysfunction	(Clarke et al., 2012; Parham et al., 2016)
		Stroke and vascular dementia	(Lee et al., 2011; Marcelo and Bix, 2015)
		Neointimal hyperplasia	(Rnjak-Kovacina et al., 2016)

PG mimetics that convey similar bioactivity as their native counterparts are gaining popularity due to larger level of control over synthesis and optimization as well as cost effectiveness. Synthetic methodology, however, has its own challenging barriers toward manufacturing commercially relevant quantities. Rapid progress in synthetic GAG synthesis and sequencing, and current understanding of kinetics of PG binding interactions with growth factors are helping scientists create the next generation of PG therapies to control and target a variety of diseases. Key features of GAG length and sulfation alongside core protein interactions are being modulated to enhance binding interactions.

Focus on targeted and controlled release of these PGs is also gaining interest. Engineering PGs to sequester and control growth factor release are being explored for enhanced therapies. Approaches to target specific tissues, such as exploiting the binding ability of core proteins to collagen, or to HA, are being explored to create localized and functional treatments. Synthetic approaches to circumvent the heterogeneity of native PGs are being employed to control and tune specific sulfation patterns, binding potential, and specificity of mimetics to establish novel ways of modulating disease state. Furthermore, chemists and cell biologists are establishing novel mimetics that don’t just necessarily mimic the structure of PGs, but also their function. There is still much to learn about the structure function relationships of PGs. Nevertheless, nascent preclinical developments have shown the promise of PG therapeutics to pioneer future treatments and breakthroughs in multiple disease indications such as wound healing, cancer, angiogenesis and hypertrophic scarring. Overall, advances in PG and GAG-based therapeutic development are putting a renewed focus on the importance of the ECM for tissue health and cell function, and opening the door for new classes of bioinspired and targeted drugs.

## Author Contributions

TW did the initial literature search and completed the first draft. AP suggested additional literature and added critical analysis and future directions.

## Funding

This work is supported in part by the National Heart, Lung, and Blood Institute and the National Institute on Drug Abuse through grant 1U54HL119893.

## Conflict of Interest

AP has licensed proteoglycan mimetic technology to Symic Bio for commercial development.

The remaining author declares that the research was conducted in the absence of any commercial or financial relationships that could be construed as a potential conflict of interest.
